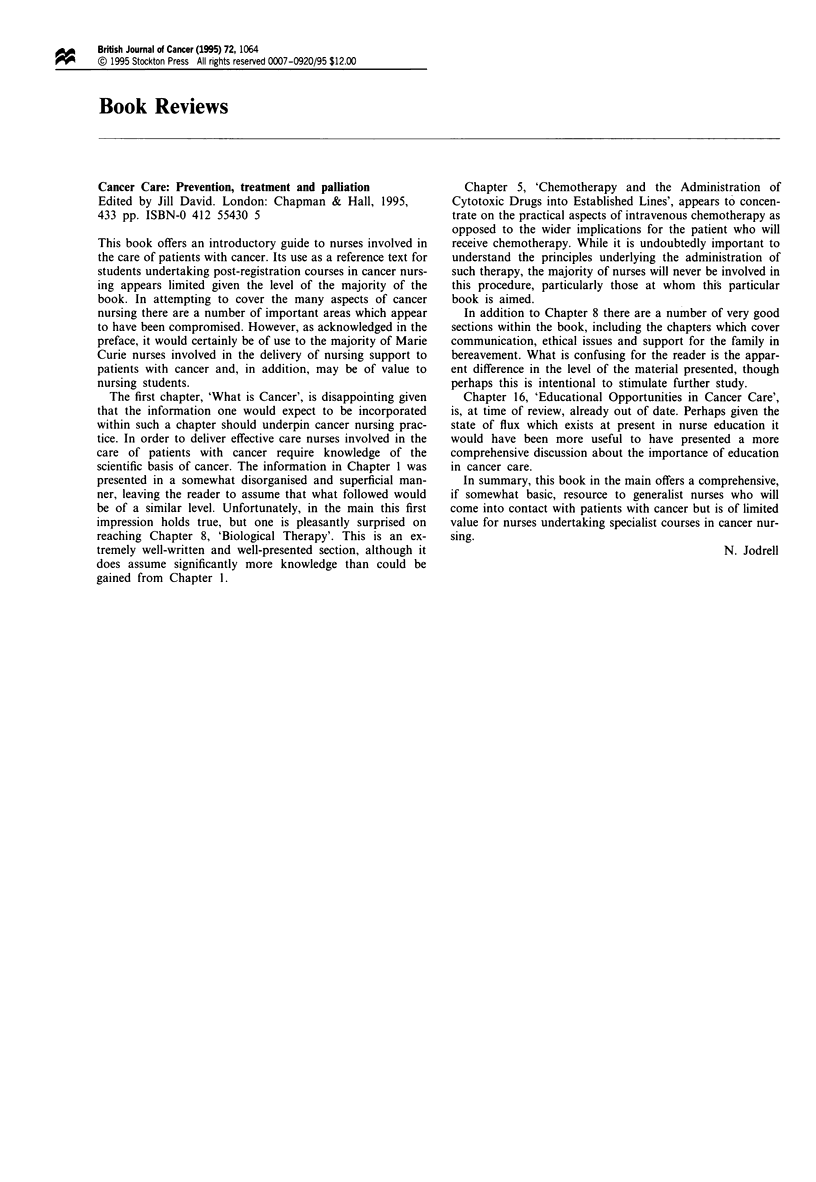# Cancer Care: Prevention, treatment and palliation

**Published:** 1995-10

**Authors:** N. Jodrell


British Journal of Cancer (1995) 72, 1064
© 1995 Stockton Press All rights reserved 0007-0920/95 $12.00

### Book Reviews

**Cancer Care: Prevention, treatment and palliation**
Edited by Jill David. London: Chapman & Hall, 1995, 433 pp. ISBN-0 412 55430 5

This book offers an introductory guide to nurses involved in the care of patients with cancer. Its use as a reference text for students undertaking post-registration courses in cancer nursing appears limited given the level of the majority of the book. In attempting to cover the many aspects of cancer nursing there are a number of important areas which appear to have been compromised. However, as acknowledged in the preface, it would certainly be of use to the majority of Marie Curie nurses involved in the delivery of nursing support to patients with cancer and, in addition, may be of value to nursing students.

The first chapter, 'What is Cancer', is disappointing given that the information one would expect to be incorporated within such a chapter should underpin cancer nursing practice. In order to deliver effective care nurses involved in the care of patients with cancer require knowledge of the scientific basis of cancer. The information in Chapter 1 was presented in a somewhat disorganised and superficial manner, leaving the reader to assume that what followed would be of a similar level. Unfortunately, in the main this first impression holds true, but one is pleasantly surprised on reaching Chapter 8, 'Biological Therapy'. This is an extremely well-written and well-presented section, although it does assume significantly more knowledge than could be gained from Chapter 1.

Chapter 5, 'Chemotherapy and the Administration of Cytotoxic Drugs into Established Lines', appears to concentrate on the practical aspects of intravenous chemotherapy as opposed to the wider implications for the patient who will receive chemotherapy. While it is undoubtedly important to understand the principles underlying the administration of such therapy, the majority of nurses will never be involved in this procedure, particularly those at whom this particular book is aimed.

In addition to Chapter 8 there are a number of very good sections within the book, including the chapters which cover communication, ethical issues and support for the family in bereavement. What is confusing for the reader is the apparent difference in the level of the material presented, though perhaps this is intentional to stimulate further study.

Chapter 16, 'Educational Opportunities in Cancer Care', is, at time of review, already out of date. Perhaps given the state of flux which exists at present in nurse education it would have been more useful to have presented a more comprehensive discussion about the importance of education in cancer care.

In summary, this book in the main offers a comprehensive, if somewhat basic, resource to generalist nurses who will come into contact with patients with cancer but is of limited value for nurses undertaking specialist courses in cancer nursing.

N. Jodrell